# Radiosynthesis, quality control, biodistribution, and infection-imaging study of a new ^99m^Tc-labeled ertapenem radiopharmaceutical

**DOI:** 10.3389/fchem.2022.1020387

**Published:** 2022-11-07

**Authors:** Syed Ali Raza Naqvi, Tania Jabbar, Maha A. Alharbi, Asma Noureen, Nada K. Alharbi, Tauqir A. Sherazi, Anum Shahzadi, Ahmed Ezzat Ahmed, M. Shahzad Afzal, M. Babar Imran

**Affiliations:** ^1^ Department of Chemistry, Government College University, Faisalabad, Pakistan; ^2^ Punjab Institute of Nuclear Medicine, Faisalabad, Pakistan; ^3^ Department of Biology, College of Science, Princess Nourah bint Abdulrahman University, Riyadh, Saudi Arabia; ^4^ Department of Zoology, Ghazi University, Dera Ghazi Khan, Pakistan; ^5^ Department of Chemistry, COMSATS University Islamabad, Abbottabad, Pakistan; ^6^ Department of Biology, College of Science, King Khalid University, Abha, Saudi Arabia; ^7^ Department of Theriogenology, Faculty of Veterinary Medicine, South Valley University, Qena, Egypt

**Keywords:** nuclear medicine, metal complex, infection imaging, radiopharmaceuticals, SPECT imaging, ertapenem

## Abstract

Ertapenem is a member of carbapenem antibiotics used for the treatment of moderate-to-severe intra-abdominal, urinary tract, acute pelvic, and post-surgical gynecologic infections. The antibacterial activity of ertapenem is mediated through binding to penicillin-binding proteins which results in inhibiting the cross-linking of the peptidoglycan layer of the bacterial cell wall. Therefore, ertapenem can be labeled with technetium-99m (^99m^Tc), a gamma emitter radionuclide, for the diagnosis of deep-seated bacterial infections, such as urinary tract, intra-abdominal, osteomyelitis, and post-surgical gynecologic infections. The labeling procedure was carried out by varying the reaction conditions, such as the amount of the ligand and reducing agent, pH, reaction time and temperature, and radioactivity. At optimized reaction conditions more than 93% ^99m^Tc–ertapenem radioconjugate was obtained. ^99m^Tc–ertapenem was found 90% intact in saline medium up to 6 h, while 88% intact in human blood serum up to 3 h. Biodistribution study showed target-to-non-target ratios of 2.91 ± 0.19, 2.39 ± 0.31, and 1.23 ± 0.22 in *S. aureus*, *E. coli*, and turpentine oil-infected rat models, respectively. The SPECT scintigraphy showed high uptake of ^99m^Tc–ertapenem in bacterial-infected abscesses, and low counts were recorded in normal and turpentine oil-inflamed tissues. In conclusion, ^99m^Tc–ertapenem can be a potent infection-imaging agent, which can diagnosis deep-seated bacterial infections at early stage but need further pre-clinical evaluation in variety of infection models.

## Introduction

Infection is a leading cause of death and illness, not only in developing nations but also worldwide. Tuberculosis and multidrug-resistant bacteria are becoming more common, posing diagnostic, therapeutic, and infection control challenges. Nuclear medicine techniques are often advised in the context of deep-seated bacterial infections and fever-of-unknown-origin to aid in accurate diagnosis and to decide about infection therapy. Initially, in the 1970s, gallium-67 (^67^Ga) was approved as a tumor-imaging agent, but soon it was found to diagnose both acute and chronic inflammation followed by the introduction of ^99m^Tc-labeled leukocytes for infection imaging. Later on, both were reported less than ideal infection-imaging agents. Existing imaging modalities, such as computed tomography (CT) scan and magnetic resonance imaging (MRI), are highly sensitive but sense only morphological changes at the disease site. A novel approach using a radiolabeled antibiotic, ^99m^Tc–ciprofloxacin, was introduced in 1995 that was approved in the early 2000s as an infection-imaging agent and marketed under the trade name Infecton^®^ with great enthusiasm (Britton et al., 2002). Due to its promising efficacy, it was reported as the gold standard infection-imaging radiopharmaceutical. The emergence of multidrug-resistant (MDR) bacteria badly hampered the imaging efficacy of Infecton^®^ (Sarda et al., 2003). However, it triggered the research on radiolabeling of different antibiotics, peptides, and small organic molecules to address the challenge of accurate diagnosis of deep-seated bacterial infections, especially urinary tract, intra-abdominal, osteomyelitis, endocarditis, and post-surgical gynecologic infections (Akhtar et al., 2004; Dahiya et al., 2009; Naqvi and Drlica, 2017).

**FIGURE 1:**
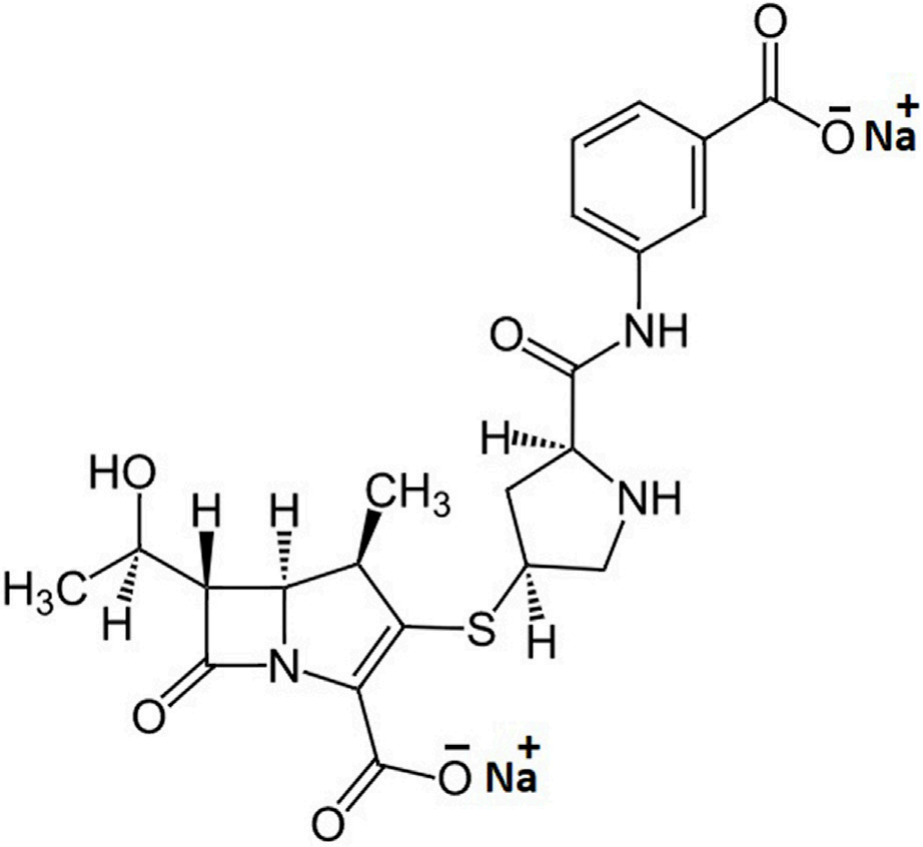
Chemical structure of sodium salt of ertapenem

Ertapenem, the chemical structure is shown in Figure 1, is a member of carbapenem antibiotics showing good efficacy against MDR bacteria; therefore, it is effectively used in the treatment of moderate-to-severe intra-abdominal, urinary tract, acute pelvic, and post-surgical gynecologic infections (Bader et al., 2019). Its efficacy profile data also reflect that the ertapenem can effectively break the additional outer membrane of Gram-negative bacteria, such as *Acinetobacter baumannii* and *Pseudomonas aeruginosa*, kill *E. coli* and *Klebsiella* spp., and shorten the hospitalization time in adults after cefotaxime-resistant Enterobacteriaceae bloodstream infections (Lee et al., 2018; Sabu et al., 2018; Song et al., 2021). The antibacterial activity of ertapenem is mediated through binding to penicillin-binding proteins (PBPs). In the case of *Escherichia coli* (*E. coli*), it firmly binds to PBP-1a, PBP-1b, and PBP-2, -3, -4, and -5 with a preference for PBP-2 and PBP-3 (Shah and Isaacs 2003). Therefore, the development of ^99m^Tc-labeled ertapenem would be an agent of choice to diagnose *E. coli-*mediated infections in particular and many other bacterial infections in the broad spectrum. Therefore, the labeling of ertapenem with the gamma-emitter radionuclide, technetium-99m, may fulfill the gap of infection-imaging using ^99m^Tc–ciprofloxacin radiopharmaceutical. ^99m^Tc is being used in more than 85% of nuclear medicine procedures due to its significant half-life (6 h) and the emission of body-compatible 140-keV gamma photons of mono-wavelength (Naqvi and Drlica, 2017; Naqvi et al., 2018; Naqvi, 2022). The key characteristics of infection-imaging agents include good stability, no particle emission, short half-life, and high target-to-non-target ratio.

Herein, we are reporting the labeling strategy of ertapenem with ^99m^Tc to introduce a new SPECT imaging agent and its biodistribution and imaging potential using *S. aureus* and *E. coli* bacterial infections induced rat and rabbit models.

## Materials and methods

### Chemicals

^99m^Tc was eluted from a Mo-99/Tc-99m PAKGEN generator obtained from the Pakistan Institute of Nuclear Science and Technology (PINSTECH), Islamabad, Pakistan. All chemicals such as stannous chloride (SnCl_2_.2H_2_O), sodium acetate, ethanol, ammonium hydroxide, trichloroacetic acid (TCA), acetonitrile, acetone, and hydrochloric acid were of analytical grade and obtained from Sigma-Aldrich, Germany. Ertapenem injection (INVANZ^®^ manufactured by MSD) in the form of sodium salt was obtained from the local pharmacy. Instant thin-layer chromatographic (ITLC-SG) sheets were purchased from Agilent Technology (Germany). Normal saline was prepared indigenously, and Milli-Q water (resistivity 18.2 MΩ cm at 25°C) was obtained from a Direct-Q system (Millipore) and used for the preparation of buffer solutions. *Staphylococcus aureus* (*S. aureus*) and *Escherichia coli* (*E. coli*) bacterial strains were obtained from the Department of Microbiology, GC University, Faisalabad, Pakistan. Albino white rats (130—150 g) and rabbits (1.25–1.5 kg), were obtained from the Department of Physiology, GC University, Faisalabad, Pakistan, to perform biodistribution and scintigraphy following the guidelines devised by the Punjab Institute of Nuclear Medicine (PINUM), Faisalabad, and the FELASA standards (Guillen, 2012).

### Radiosynthesis of ^99m^Tc–ertapenem

Radiosynthesis of ^99m^Tc–ertapenem was carried out by varying the reaction conditions, such as ligand concentration, reducing agent concentration, pH, and reaction time to obtain maximum radiochemical yield. However, radioactivity and temperature were kept constant. In order to obtain the maximum and stable radiochemical yield, ertapenem was labeled with freshly eluted ^99m^TcO_4_^–^. The optimization of reaction parameters was performed by studying the effect of the ligand concentration (0.25—1.5 mg/ml), SnCl_2_•2H_2_O (5—30 μg/ml), and pH (9–11). All the reagents were mixed and vortexed in a glass vial followed by the addition of ∼2 mCi saline solution of pertechnetate. The reaction mixture was then incubated for different time intervals (5—30 min) at room temperature.

### Chromatography analysis of the radiochemical mixture

**FIGURE 2:**
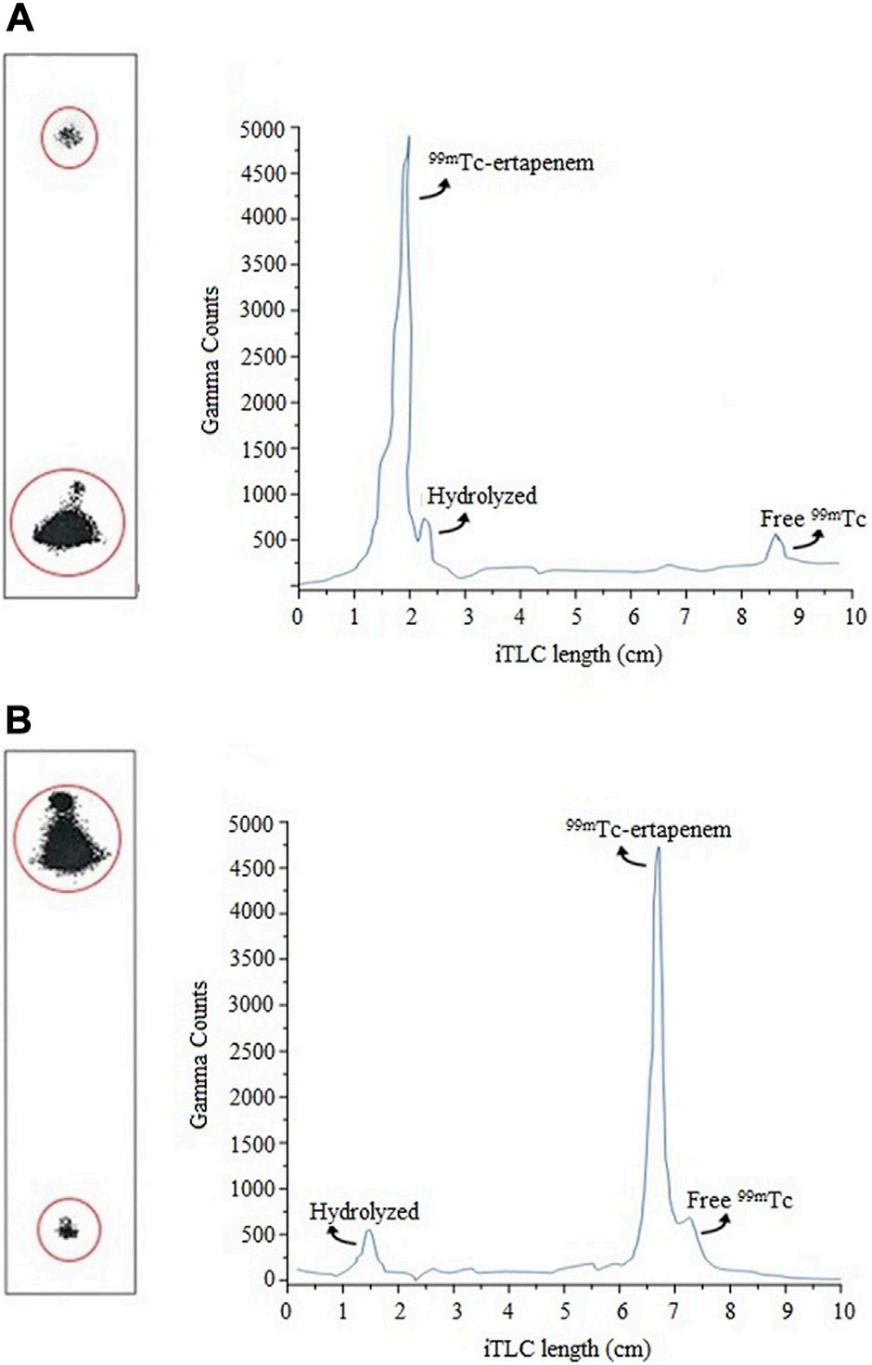
Radio-chromatograms of radiochemical mixture analysis using ITLC: **(A)** determination of free ^99m^Tc; **(B)** determination hydrolyzed radioactive impurity.

The radiochemical labeling yield was analyzed by instant thin-layer chromatography impregnated with a silica gel (ITLC-SG) technique. A small aliquot, ∼5 μL of the reaction mixture, was spotted at the baseline of the ITLC-SG strip. The radiochemical mixture was allowed to develop with an acetone mobile phase, which results in free ^99m^Tc elution to the solvent front, leaving the hydrolyzed ^99m^Tc and bound ^99m^Tc at the baseline. The radiochromatography analysis is shown in Figure 2A. The counts were recorded by cutting the paper into small segments (0.25 cm) using an NaI(Tl) gamma counter. The free ^99m^Tc was then calculated by using Equation 1 as follows:

$${Free}^{99m}Tc\left(\%\right)=\frac{{Radioactivity\;Counts\;at\;R}_{f}\;0.75-1}{{Total\;Radioactivity\;Counts\;at\;R}_{f}\;0-1}.$$ (1)

To determine the hydrolyzed impurity, an aliquot of ∼5 µL reaction mixture was spotted at the baseline of the ITLC-SG strip and allowed to develop in a mixture of ethanol, water, and ammonium hydroxide (2:5:1) solution with a mobile phase. In this system, the mobile phase picks ^99m^Tc–ertapenem and free ^99m^Tc to the solvent front, leaving hydrolyzed ^99m^Tc at the baseline. The counts were recorded by cutting the paper into small segments (0.25 cm) using the NaI(Tl) gamma counter. The radiochromatography analysis is shown in Figure 2B. The percentage of hydrolyzed ^99m^Tc was calculated using Equation 2 as follows:

$$Hydrolyzed\left(\%\right)=\frac{{Radioactivity\;counts\;at\;R}_{f}\;0-0.25}{Total\;radioactivity\;counts\;on\;ITLC\;strip}.$$ (2)

Finally, the percent yield of ^99m^Tc–ertapenem was determined by using Equation 3 as follows:

T99mc−ertapenem yield(%)=100−(free99mTcO4−+hydrolized99mTc).

### Stability of the radioconjugate in saline

Post-labeling stability of the ^99m^Tc–ertapenem radiocomplex was analyzed in saline and freshly harvested human blood serum medium up to 6 h at room and physiological temperatures, respectively. The reaction mixture of >93% ^99m^Tc–ertapenem was incubated at room temperature followed by assessing the stability of the radiocomplex at different time points, such as 0.5, 1.5, 3, and 6 h, by ITLC-SG chromatography.

### Stability of the radioconjugate in blood serum

Fresh human blood was collected from a healthy subject. The blood was centrifuged at 3,000 rpm for 10 min which separated the serum as a supernatant fraction that was collected in a separate vial. In a sterilized cryotube, 200 µL of freshly radiolabeled reaction mixture showing >93% ^99m^Tc–ertapenem was mixed with 1.8 mL of serum, vortexed for 30 s, and finally incubated at 37°C in a CO_2_ incubator. At post-0.5 h, -1.5 h, -3 h, and -6 h incubating periods, an aliquot of 10 µL was withdrawn and spotted at the baseline of the ITLC-SG strips to analyze intact ^99m^Tc–ertapenem and radioactive impurities.

### Lipophilicity studies of the radioconjugate

The lipophilicity characteristics of the ^99m^Tc–ertapenem radioconjugate were determined by calculating the logarithm of the distribution coefficient (logD) in n-octanol/phosphate-buffered solution (PBS) of pH 7.40. The study was carried out by adding 1 ml ^99m^Tc–ertapenem radioconjugate, previously filtered through a 0.22-mm Millipore filter to get rid of hydrolyzed radioactive impurities, to a test tube containing 1 ml n-octanol for the logP calculation or mixing 100 µL with 900 µL PBS followed by mixing with 1 ml of n-octanol for the logD calculation. The mixtures were then vortexed for 1 min followed by centrifugation for 5 min at 6,000 rpm to ensure complete separation of the radioconjugate into two layers. The two layers were then collected separately, and the gamma counts were recorded using a well-type NaI(Tl) detector. After adjusting for free ^99m^Tc counts and the ratio of the activity of the organic to that of the aqueous phase (LO/LA), the logP and logD values were calculated.

### Biodistribution in infected rats

The biodistribution and excretory route of ^99m^Tc–ertapenem was studied in albino white rats (n = 3 for each group) divided into *S. aureus*- and *E. coli* infection-induced rats and turpentine oil inflammation-induced rats following the procedure reported in the literature (Naqvi et al., 2012). Post-30 h period of introducing infection and inflammation, when clear redness and swelling were noticed, the animals were injected with 100 μL of ^99m^Tc–ertapenem (∼1 mCi) intravenously through the tail vein. After chloroform anesthesia and dissection, the organs of interest, such as the kidneys, liver, heart, spleen, lungs, stomach, intestine, and inflamed and infected muscle, were removed, washed using saline, weighed, and radioactivity counts in each organ were measured using a gamma counter. Measured counts were adjusted to the initial time with the respective decay of ^99m^Tc isotope, and the data were expressed in terms of the percent injected dose per gram (%ID/g) body organ.

### Whole-body scintigraphy studies

Gamma scintigraphy was performed using a single-headed Siemens gamma camera (E.CAM). New Zealand white rabbits (weight 1.5–2 kg) were used in whole-body scintigraphy studies. Rabbits in group A (n = 3) were infected with *S. aureus* using 150 μL of 1 × 108 colony-forming units (CFU) in right thigh muscles, and inflammation was induced in left thigh muscles using 150 μL sterile turpentine oil; in group B (n = 3), animals were infected with *E. Coli* keeping all parameters similar to group A. Group C (n = 3) was designed as the control group consisting of healthy animals to record the normal biodistribution of the ^99m^Tc–ertapenem radiocomplex and free ^99m^Tc. In group D (n = 3), the *S. aureus* bacterial infection was induced in the right thigh muscles, while *E. Coli* bacterial infection was induced in the left thigh muscles. Physical observation (swelling and redness in infected muscles) and blood tests of all infected groups were carried out to confirm the infections. An aliquot of 250 µL of ^99m^Tc–ertapenem radiocomplex (2 mCi) was injected into the marginal ear vein for recording specific and non-specific accumulation, washout period, and renal filtration. The animal was then anesthetized by diazepam injection intramuscularly using the dose limit of 20 mg/kg. The anesthetized animal was placed in the supine position to obtain anterior and posterior whole-body images at post-5 min, -30 min, -2 h, and -4 h post-injection period.

## Results

### Physical characteristics

The physical appearance of the labeled ertapenem mixture was noted to be a colorless and odorless transparent solution. In the reaction mixture, no particulate matter was observed. Traces of colloids formed were removed by passing the radiochemical mixture through a 0.22-µm filter.

### Effect of quality control parameters on labeling yield

The effects of the ligand, reducing agent, pH, and reaction time were studied in the development of ^99m^Tc–ertapenem radiopharmaceutical, to obtain maximum radiochemical yield and minimum radioactive impurities.

### Effect of ligand concentration

**FIGURE 3:**
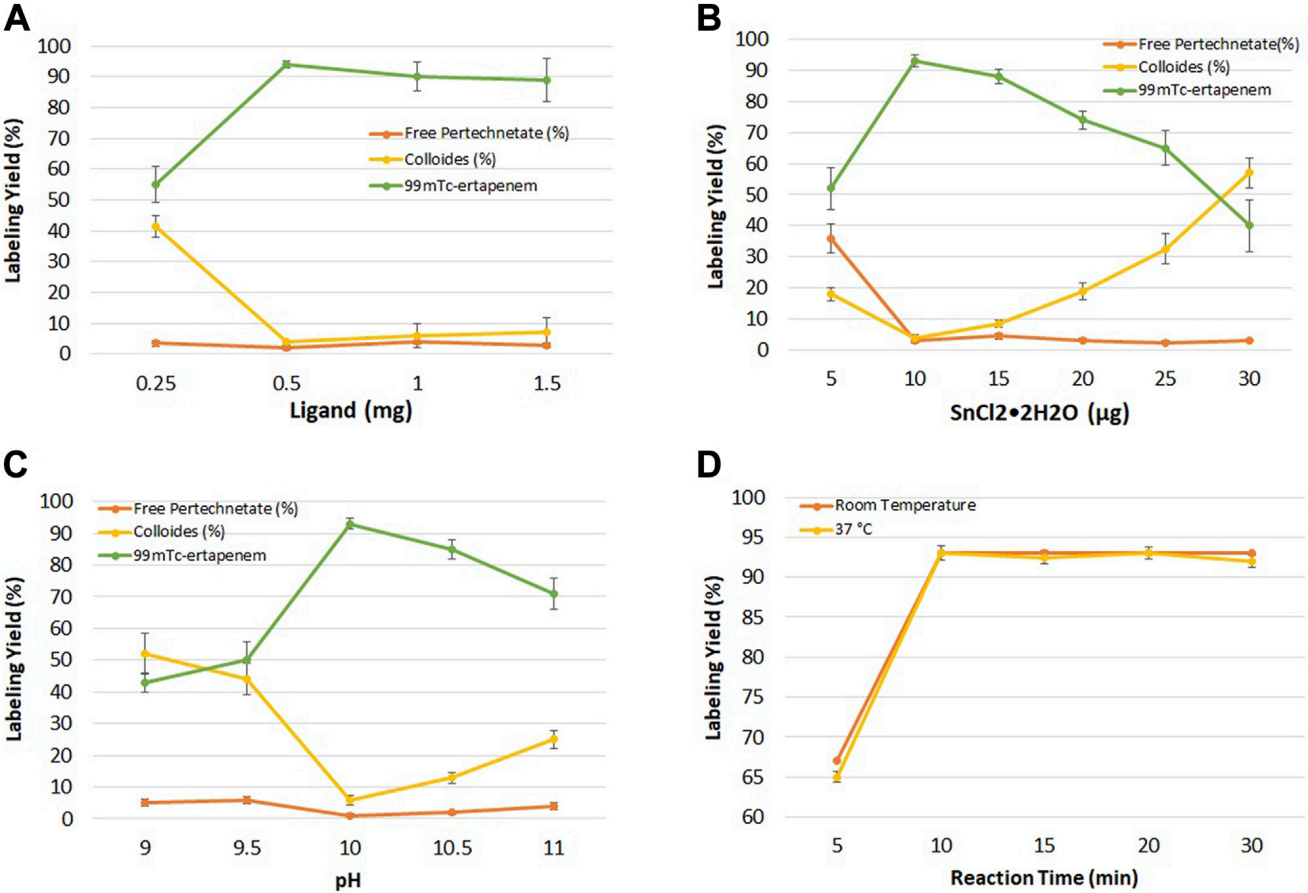
**(A)** Effect of ligand (ertapenem) concentration on radiochemical yield; **(B)** effect of the reducing agent (SnCl_2_•2H_2_O) on radiochemical yield; **(C)** effect of pH on radiochemical yield; and **(D)** effect of reaction time on radiochemical yield.

Figure 3A shows the effect of ertapenem concentration on radiochemical yield. The ligand concentration was studied in the range of 0.25–1.5 mg at an interval of 0.5 mg concentration. The maximum yield was obtained at 0.5 mg/ml concentration. At higher concentrations, no significant change in radiolabeling was recorded.

### Effect of the reducing agent

Figure 3B shows the summary of the effect of reducing agents on radiolabeling yield. In the pre-radiolabeling step, the reduction of ^99m^Tc was carried out with SnCl_2_•2H_2_O, which is commonly used to reduce ^99m^Tc from its higher oxidation state to a lower one in ^99m^Tc radiopharmaceutical developments. The effect of SnCl_2_•2H_2_O concentration on radiochemical yield was studied in the range of 5–30 μg/ml, keeping other parameters constant.

### Effect of pH

The effect of hydrogen ion concentration in keeping the radiochemical stable and soluble was investigated at basic pH. Figure 3C shows the results of the pH effect on radiochemical yield. Maximum yield was obtained at pH 10 which decreased to 70% at pH 11.

### Effect of the reaction time

The effect of the reaction time on the radiolabeling yield was evaluated at room temperature for different time intervals (5–30 min). Figure 3D shows the effect of the reaction time on radiochemical yield. More than 93% radiochemical yield was recorded when the reaction was performed for up to 10 min at room and physiological temperature.

### Optimized radiolabeling reaction conditions

**SCHEME 1:**
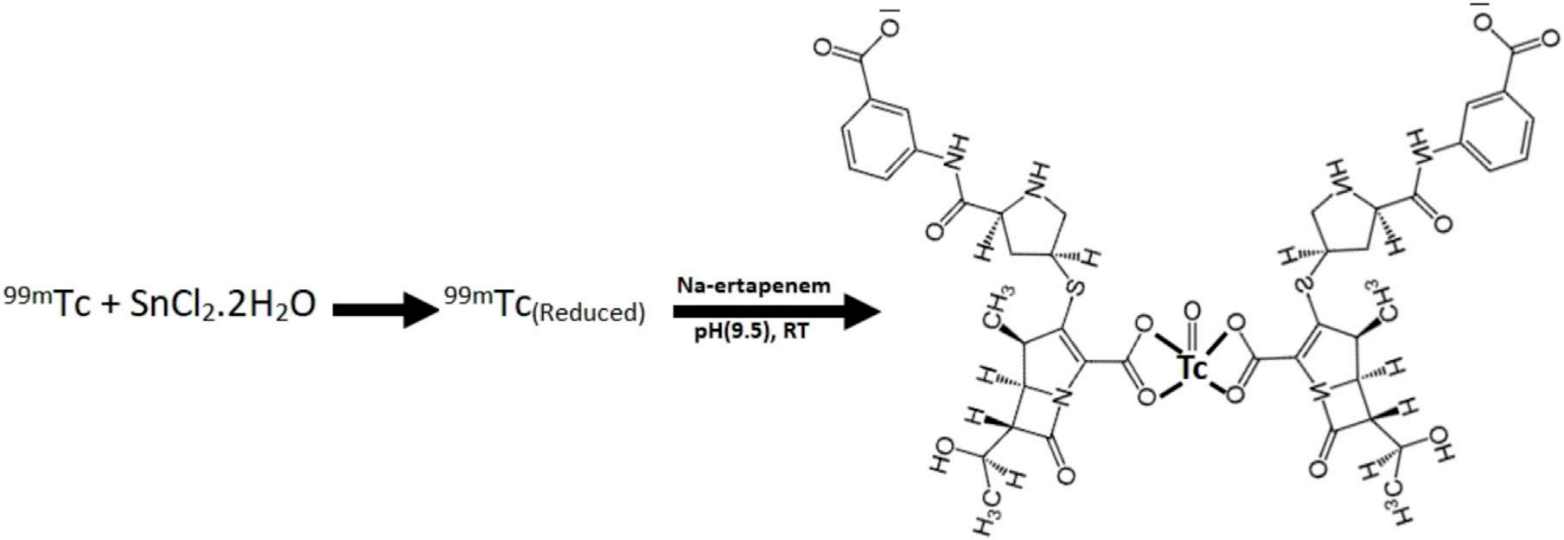
Radiosynthesis scheme with the predictive chemical structure of ^99m^Tc–ertapenem.

Maximum radiochemical yield (93%) was obtained when 0.5 mg/ml ertapenem, 10 µg SnCl_2_•2H_2_O, and 2 mCi ^99m^TcO4-1 were mixed and the pH of the mixture was adjusted to 10 using 0.05 N NaOH or HCl at room temperature for 10 min in a total reaction volume of 2 mL. The radiosynthesis scheme with the predicted chemical structure of ^99m^Tc–ertapenem conjugate is shown in Scheme 1.

### Stability and lipophilicity studies of the ^99m^Tc–ertapenem radioconjugate

**FIGURE 4:**
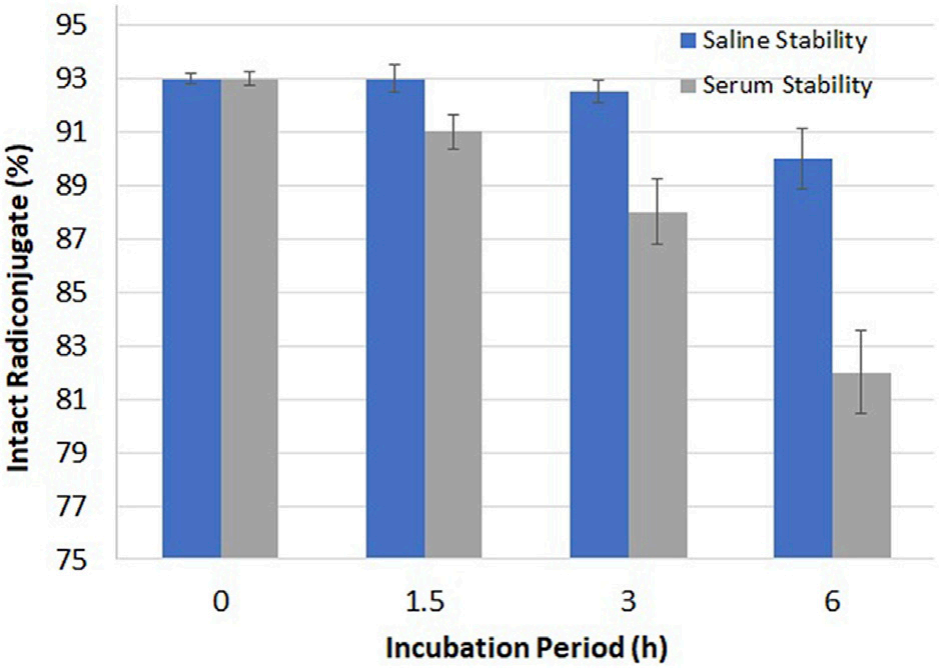
Stability of ^99m^Tc–ertapenem in saline and blood serum.

The stability of ^99m^Tc–ertapenem was examined in saline and blood serum using quality control analysis as shown in Figure 4. At post 3 h of the incubation period, 92.45 and 88.67% intact radiochemicals were recorded in the saline and serum media, respectively. However, at 6 h post-incubation period, these values were 90.35 and 82.42%, respectively. The radioconjugate was transferred more to the organic phase in both experiments. The logD value was recorded as -1.17 ± 0.12.

### Biodistribution study of ^99m^Tc–ertapenem

**FIGURE 5:**
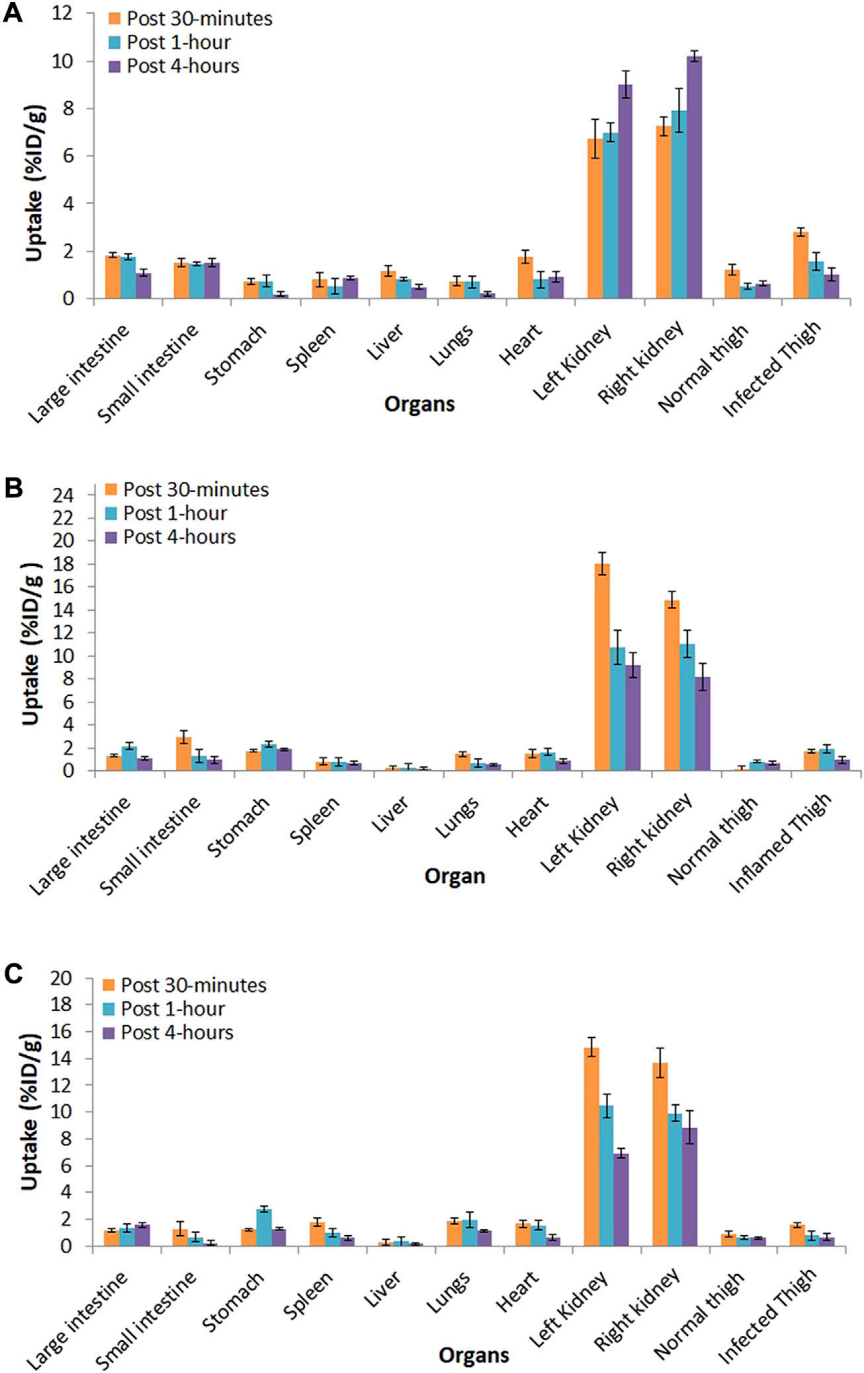
Biodistribution of ^99m^Tc–ertapenem in; **(A)** *S. aureus* infection induced rat models, **(B)** *E. coli* infection induced rat models and, **(C)** inflammation induced rat models.

The biodistribution of ^99m^Tc–ertapenem was studied in infection- and inflammation-induced albino (*Rattus norvegicus*) rats at three different time points, namely, 30 min, 1, and 4 h. Figure 5A shows the distribution in *S. aureus* infection-induced rat models, Figure 5B shows the distribution in *E. coli* infection-induced rat models, and Figure 5C shows the distribution in turpentine oil inflammation-induced rat models. The data show the radiochemical uptake in various organs and washout behavior. However, in parallel, positive and negative control groups were also studied to record the precise biodistribution and the uptake of bound and free ^99m^Tc in different body tissues.

### Scintigraphy study

**FIGURE 6:**
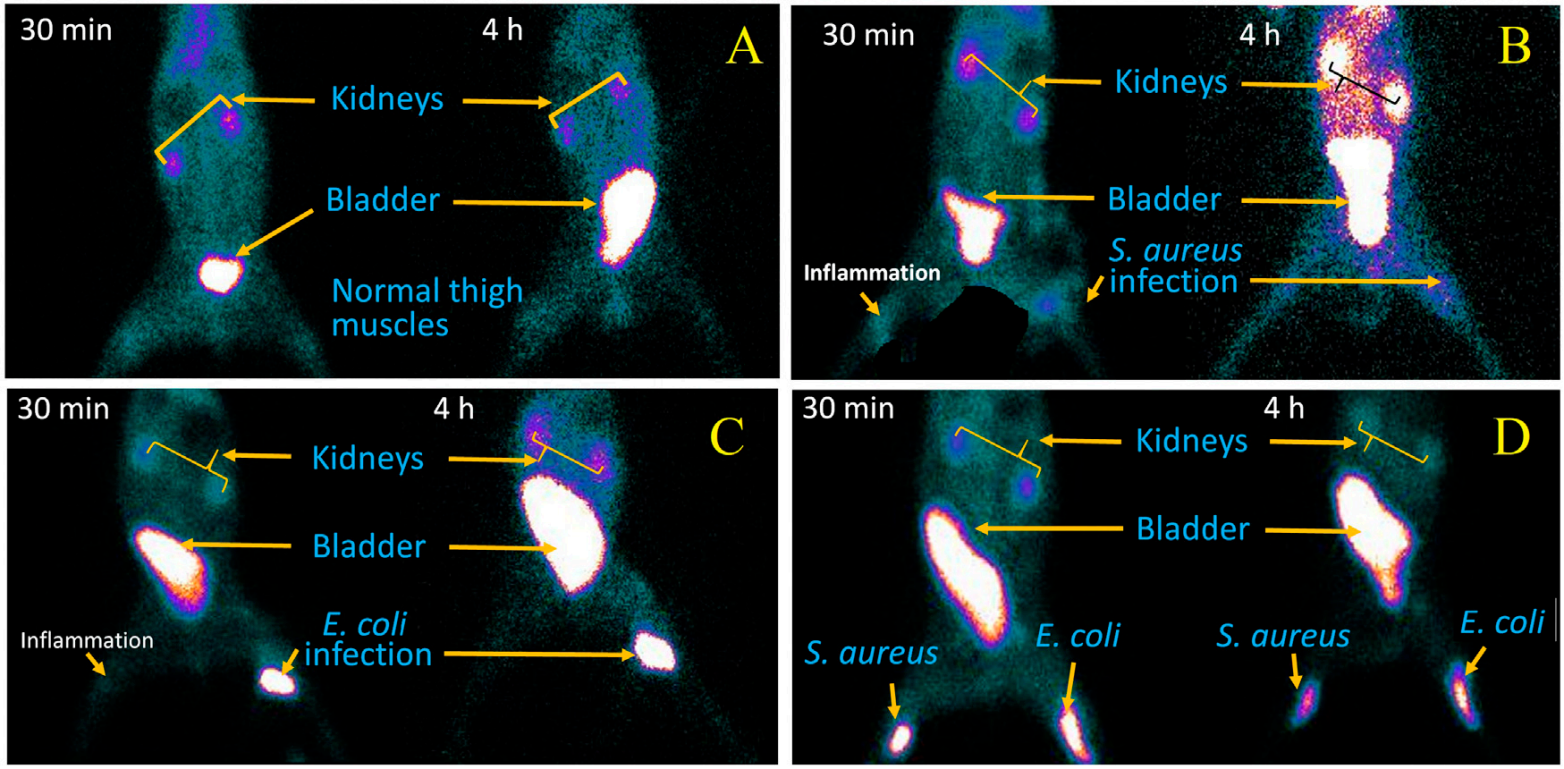
SPECT gamma camera scintigraphy images, at 30 min and 4 h post injection of ^99m^Tc-ertapenem radiopharmaceutical, of: **(A)** healthy rabits, **(B)** *S. aureus* infection and inflammation induced rabits, **(C)** *E. coli* infection and inflammation induced rabits and, **(D)** *S. aureus* and *E.coli* infection induced rabits.

A scintigraphy study was performed on ^99m^Tc–ertapenem-administered rabbit models to record the biodistribution and accumulation in infected, inflamed thigh muscles and different body organs; the post-accumulation activity washout period from target and non-target organs and renal filtration were carried out using a SPECT gamma camera. The rabbit models were designed similar to rat models. Anterior and posterior whole-body planar images of ^99m^Tc–ertapenem-administered rabbits in the supine position were recorded at 5 min, 30 min, 2 h, and 4 h. The scintigraphy images at different time intervals are shown in Figure 6.

## Discussion

Targeted diagnosis and therapy is an open debate in clinical practice to address infectious and malignant diseases in the shortest possible period. Precise diagnosis and timely therapy of MDR bacterial infections can effectively reduce the rate of mortality and morbidity in the recent era. Antibiotics are agents that manage bacterial infections; however, due to the emergence of MDR bacteria, the maximum number of antibiotics shows deficits in infection treatment efficacy (Ventola, 2015; Xia and Jiang, 2020). This phenomenon had also affected the clinical infection-imaging efficacy of ^99m^Tc–ciprofloxacin and many other ^99m^Tc-labeled antibiotics (Sarda et al., 2003). This study is an attempt to develop ^99m^Tc-labeled ertapenem due to its high efficacy against MDR bacteria. The ^99m^Tc–ertapenem radioconjugate was evaluated for its physiochemical, biological, and infection diagnosis potential. Under optimized reaction conditions, more than 87% radiochemical was obtained which further increased to 93–94% post-filtration process through a 0.22-µm Millipore filter. The final radiochemical was found to be 90% intact in a saline medium up to 6 h. The radiochemical yield, we obtained, was in good agreement with ^99m^Tc–ciprofloxacin radioconjugate yield and radiopharmaceutical purity standards, required for clinical practice. The radioconjugates with less than 90% radiochemical purity, in most nuclear medicine procedures, are not recommended to be administered into a patient’s body. In developing new radiopharmaceuticals with maximum yield and stability; typically, the optimum conditions such as ligand concentration, reducing agent concentration, pH, and reaction time are investigated. The reducing agent and pH, however, decide the yield and stability of the radioconjugate. SnCl_2_•2H_2_O was used as an reducing agent that allows the reduction of oxidation state of ^99m^Tc (from +7) to a lower one. Commonly, compatible to make a complex with ertapenem. Other reducing agents have also been reported in the literature in ^99m^Tc-labeling chemistry, for example, formamidine sulfinic acid (FSA), stannous citrate, and stannous tartrate*.* SnCl_2_•2H_2_O is considered the best reducing agent due to its stability and integrity at high temperatures which allows us to investigate labeling reactions at a broad range of temperatures. FSA is an organic acid and less toxic than metallic stannous salts; however, due to its decomposition upon heating and imparting yellow color to the reaction mixture, it is not frequently used in ^99m^Tc chemistry (Oh et al., 2002). Furthermore, a minute amount of SnCl_2_•2H_2_O is sufficient to reduce a high amount of ^99m^Tc. Here, in this study, 10 μg/mL was found to be a good concentration to reduce 2 mCi ^99m^TcO_4_-1. Above this amount, Sn^+2^ ions were hydrolyzed to colloidal particles, when a low ligand concentration was used at basic pH. In common practice, SnCl_2_•2H_2_O is taken about 1,000–1,000,000 times more than ^99m^TcO_4_-1 activity. It eliminates the chance of the presence of non-reduced free ^99m^TcO_4_-1. In the radiochemical synthesis reaction, pH plays a critical role in stabilizing the coordinate covalent bonds of the complex which, otherwise, is not possible to make it stable. At an optimized pH (9.5), the reaction shows >93 ± 1.67% radiochemical yield. Ideally, the physiological *p*H is the most optimal to synthesize radiopharmaceuticals, but due to the blood’s high buffer capacity, a broad range of *p*H at very low radiopharmaceutical agent administering volumes can be affordable (Iqbal et al., 2018; Saha, 2018). Under optimized reaction conditions, the stability of radiopharmaceuticals indicates that the carboxyl group from two ertapenem molecules could coordinate with ^99m^Tc, mimicking the predicted structure of ^99m^Tc–ciprofloxacin (Fang et al., 2020). According to the literature review, for most ^99m^Tc-labeled compounds used clinically, including the fluoroquinolones, the technetium (V or III)-oxo core (TcO^+3^ and TcO^+2^) in which technetium-99m shows the +5 or +3 oxidation state (respectively), is involved and stabilized through a tetradentate ligand or through a pair of bidentate ligands (Jones and Davison, 1982; Drevensek et al., 2005). The instrumental characterized structure of ^99m^Tc–ciprofloxacin and other ^99m^Tc-labeled antibiotics is still un-resolved; however, predictive structures have been reported continuously (Fang et al., 2020; Koźmiński et al., 2021). The ertapenem also carries two bidentate carboxyl groups, one at the phenyl ring and second at the azoline ring, and it is predicted that two ertapenem molecules are involved in making the complex with a TcO^+3^ or TcO_2_^+^ core, either involving the phenyl ring or the azoline ring carboxylate group. We, in this manuscript, have presented the ^99m^Tc-ertapenem structure using the azoline ring carboxylate group (Scheme 1). The stability of the complex has been tested in saline and serum media at the reaction pH and physiological temperature, resulting in good stability.

The radiochemical stability in the serum medium under physiological conditions reveals more than 90% intact radiochemical for up to 2 h, which decreases to 88.67% at 3 h and 81.52% after 6 h. The results are encouraging because scintigraphy in animal models shows maximum target accumulation and rapid washout from non-targeted organs within 1 hour of administration of radiopharmaceuticals. Therefore, the chance of radiotoxicity is minimal. The lipophilicity parameter, log *D*, showed a -1.17 ± 0.12 value at the labeling pH, which predicts that the radioconjugate is more lipophilic in nature and may show plasma binding, lipid solubility, permeability, and adsorption. The permeability through the cell membrane (lipid bi-layer) can facilitate the radioconjugate to pass through the lipid bi-layer and can bind PBPs to firmly accumulate in infected cells.

The biodistribution and clearance of ^99m^Tc–ertapenem from normal, inflamed, and infected rats after intravenous injection reflect the rapid accumulation of ^99m^Tc–ertapenem in different organs. The rate of localization of a radiopharmaceutical in an organ is related to its rate of plasma clearance after administration. The kidneys and urinary bladder showed substantial accumulation of ^99m^Tc–ertapenem due to excretory passage. According to pharmacokinetics data of ertapenem, 80% of the total administered dose of ertapenem excretes through the renal passage. Compared to infected muscles, inflamed muscles showed significantly less uptake. The small uptake at the inflammation site might be due to the over-circulation of the blood pool or secondary infection produced in inflamed muscles. A high uptake was recorded in *S*. *aureus-*infected thigh muscles (T/NT = 2.9 ± 0.19) followed by *E. coli* (T/NT = 2.39 ± 0.31) and inflamed tissues (T/NT = 1.29 ± 0.22). Compared to ^99m^Tc–gemifloxacin (2.57 ± 0.84), ^99m^Tc–ceftriaxone (2.24 ± 0.23), ^99m^Tc–clindamycin (2.37 ± 0.5), and ^99m^Tc–enrofloxacin (2.84 ± 0.63), the T/NT value of ^99m^Tc–ertapenem is high in both *S. aureus*- and *E. coli-*infected rat models. Soon after radiopharmaceutical administration, the non-targeted organs also showed accumulation of ^99m^Tc–ertapenem, mainly due to blood pool activity; however, it washed out rapidly. Scintigraphy of normal, infected, and inflamed rabbit models is shown in Figure 6. The control group showed renal filtration to the bladder at 5 min post-administration, but none of the other organs showed a significant uptake. *S. aureus* infection-induced rabbit models showed renal filtration subsequently after intravenous administration of radiochemicals; however, accumulation in *S. aureus-*infected thigh muscles was noted at 30 min in the post-administration scintigraphy image (Figure 6B). A similar mode of accumulation was also noted in the case of *E. coli-*induced rabbit model scintigraphy (Figure 6C). In both rabbit models, mild counts were detected in inflamed thigh muscles which is commonly reported due to secondary infection. The group D animal model (Figure 6D) shows renal filtration of radioactivity soon after radiopharmaceutical administration and gradual accumulation of ^99m^Tc–ertapenem in infected muscles. In all rabbit models, a slight accumulation of the radiochemical was also noted in the liver and stomach; however, rapid washout of radioactivity from non-targeted organs, rapid blood clearance, and fast renal filtration indicate body-compatible pharmacokinetics that are needed for good radiopharmaceuticals for infection diagnosis. The scintigraphy results were found to be consistent with biodistribution results. The overall results, we obtained, in this study are in good agreement with previously reported ^99m^Tc-labeled radiopharmaceuticals.

## Conclusion

Ertapenem, which bears a good profile against MDR bacteria, shows good radiolabeling yield and stability in the reaction medium and blood serum. ^99m^Tc–ertapenem showed significantly higher uptake and accumulation at the site of infection than in inflammation tissues. Therefore, due to the stability, physiochemical characteristics, biodistribution, and SPECT infection-imaging study, ^99m^Tc–ertapenem could be considered a strong candidate as an infection-imaging agent to fulfill the gap of ^99m^Tc-WBC and ^99m^Tc–ciprofloxacin for infection imaging after further pre-clinical and clinical studies, and FDA approval.

**Acknowledgement**
1) Princess Nourah bint Abdulrahman University Researchers Supporting Project Number (PNURSP2022R182), Princess Nourah bint Abdulrahman University, Riyadh, Saudi Arabia
2) The authors are grateful to the deanship of scientific research at King Khalid University, Abha, Saudi Arabia for supporting this work under the grant number (R.G.P2/117/43).
3) Higher Education Commission (HEC), Islamabad, for funding this project (No. 5612/Punjab/NRPU/R&D/HEC/2016) and the Punjab Institute of Nuclear Medicine (PINUM), Faisalabad, for providing hot lab/gamma camera facilities and technical assistance to complete this study.

## Data Availability

The original contributions presented in the study are included in the article/Supplementary Material; further inquiries can be directed to the corresponding author.
